# Canagliflozin ameliorates renal oxidative stress and inflammation by stimulating AMPK–Akt–eNOS pathway in the isoprenaline-induced oxidative stress model

**DOI:** 10.1038/s41598-020-71599-2

**Published:** 2020-09-04

**Authors:** Raquibul Hasan, Shoumen Lasker, Ahasanul Hasan, Farzana Zerin, Mushfera Zamila, Faisal Parvez, Md. Mizanur Rahman, Ferdous Khan, Nusrat Subhan, Md. Ashraful Alam

**Affiliations:** 1 Department of Pharmaceutical Sciences, College of Pharmacy, Mercer University Health Sciences Center, Mercer University, Atlanta, GA 30341 USA; 2 Department of Pharmaceutical Sciences, North South University, Bashundhara, Dhaka, 1229 Bangladesh

**Keywords:** Biochemistry, Molecular biology

## Abstract

Diabetes is a leading cause of chronic kidney disease, and the high prevalence of sympathetic nervous system (SNS) hyperactivity in diabetic patients makes them further susceptible to SNS-mediated oxidative stress and accelerated kidney damage. Here, we investigated if canagliflozin can reverse isoprenaline (ISO)-induced renal oxidative damage in rats, a model that mimics SNS overstimulation-induced organ injuries in humans. We found that ISO administration elevates renal oxidative stress markers including malondialdehyde (MDA), advanced protein oxidation product (APOP), myeloperoxidase (MPO) and nitric oxide (NO), while depleting levels of endogenous antioxidants such as catalase (CAT), superoxide dismutase (SOD) and glutathione (GSH). Strikingly, canagliflozin treatment of ISO-treated rats not only prevents elevation of oxidative stress markers but also rescues levels of depleted antioxidants. Our results also show that canagliflozin stimulates antioxidant/anti-inflammatory signaling pathways involving AMP-activated protein kinase (AMPK), Akt and eNOS, and inhibits iNOS and NADPH oxidase isoform 4 (NOX4), all of which are associated with oxidative stress and inflammation. Further, canagliflozin prevents ISO-induced apoptosis of kidney cells by inhibiting Bax protein upregulation and caspase-3 activation. Histological examination of kidney sections reveal that canagliflozin attenuates ISO-mediated increases in inflammatory cell infiltration, collagen deposition and fibrosis. Finally, consistent with these findings, canagliflozin treatment improves kidney function in ISO-treated rats, suggesting that the antioxidant effects may be clinically translatable.

## Introduction

Diabetic kidney disease is a major risk factor for the development of chronic kidney disease affecting approximately 40% of global diabetic population^1^. Diabetic kidney disease is associated with vascular inflammation, loss of renal vascular integrity and hypertension, leading to a progressive loss of renovascular function and renal failure^1^. Importantly, there is a high prevalence of sympathetic nervous system (SNS) hyperactivity in diabetic patients associated with autonomic neuropathy and concomitant vagal impairment, making diabetic patients twice as likely to develop hypertension^2^. Diabetic patients are also highly susceptible to chronic kidney disease due to renal oxidative damage and inflammation^2^. High SNS drive stimulates β1 adrenergic receptors (β1-AR) in juxtaglomerular cells, increasing renin secretion and subsequent activation of the renin–angiotensin–aldosterone system (RAAS). RAAS creates a feed-forward mechanism that accelerates renovascular dysfunction and kidney damage through various mechanisms including oxidative stress, inflammation, fibrosis, hyperproliferation, increased renal vascular resistance and hypertension^3,4^. In addition, SNS-mediated β2-AR activation stimulates NADPH oxidase-mediated reactive oxygen species (ROS) generation^5^, and activation of β2-AR in the kidney can lead to oxidative stress, inflammation and fibrosis, subsequently leading to kidney damage. Unfortunately, treatment for SNS hyperactivity in diabetes is limited because of life-threatening side effects associated with conventional beta-blocker therapy. Therefore, antidiabetic drugs which protect against SNS overstimulation, oxidative stress and inflammation are expected to reduce the overall risk of cardiovascular and kidney diseases in diabetic patients.

Accumulating evidence suggests that the antidiabetic drug canagliflozin, which is a sodium-glucose cotransporter-2 (SGLT2) inhibitor, has remarkable antioxidant and anti-inflammatory actions that reduce pathological remodeling of heart and kidneys, arterial stiffness and systemic blood pressure^6,7^. A recent study demonstrated that canagliflozin protects against cisplatin-induced nephrotoxicity in mice by reducing levels of ROS and reactive nitrogen species (RNS), while enhancing the activity of endogenous antioxidants such as superoxide dismutase (SOD) and catalase (CAT)^8^. Canagliflozin has also been shown to be effective against cardiac and renal ischemia–reperfusion injury (IRI), but ineffective in oxalate-induced nephrolithiasis in mice and nephrectomized rats^9,10^.

The promising protective effects of canagliflozin against cardiac and renal oxidative stress as well as inflammation, led us to hypothesize that canagliflozin may have a broad range of antioxidant and anti-inflammatory properties against oxidative kidney damage, including that caused by SNS hyperactivity. Therefore, in the present study we generated a model of chronic SNS hyperstimulation by administering the non-selective β-AR agonist isoprenaline (ISO) that elicits renal injury via ROS/RNS production (and resultant oxidative stress, inflammation and apoptosis)^11–13^, and investigated the effects of canagliflozin on underlying molecular mechanisms. Our data demonstrates that canagliflozin improves kidney function markers in ISO-treated rats by stimulating multiple antioxidant, anti-inflammatory and anti-apoptotic signaling pathways involving AMPK, Akt and eNOS. Together, these effects prevent ISO-induced oxidative stress caused by iNOS, NOX4, Bax and caspase-3 activation. Our study, for the first time, uncovers a novel mechanism for canagliflozin in reducing oxidative kidney damage, and raises the possibility of potential benefits of canagliflozin treatment in SNS hyperactivity-associated kidney injury in diabetes.

## Results

### Canagliflozin treatment ameliorates ISO-induced renal oxidative stress

**Figure 1:**
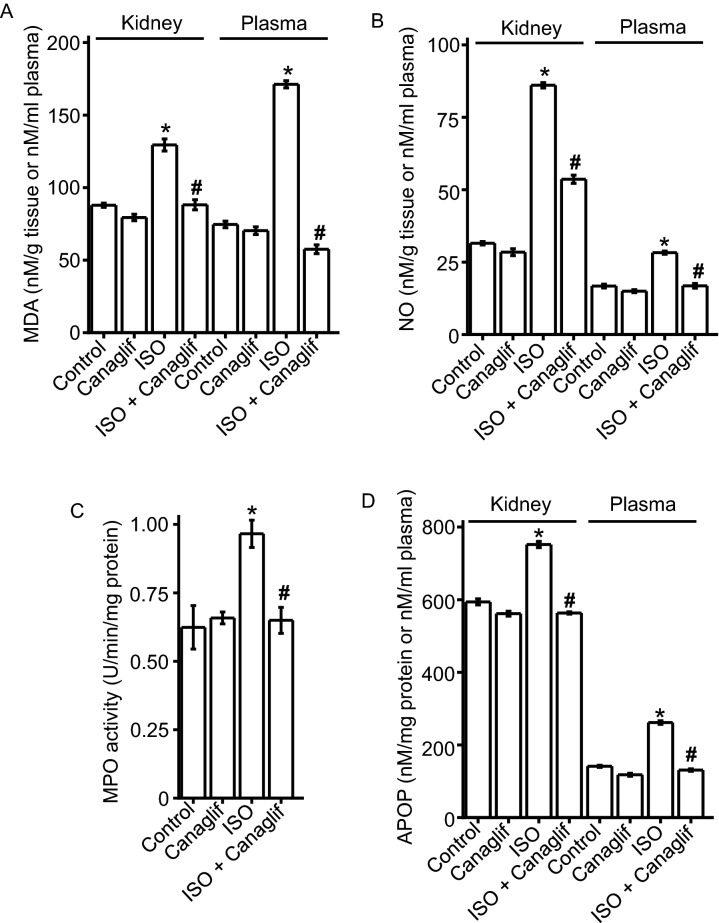
Canagliflozin (Canaglif) treatment reduces ISO-induced elevation of oxidative stress markers in kidney and plasma. One-way ANOVA with Newman–Keuls post hoc test was used for multiple group comparisons. Data are presented as mean ± SEM. n = 5 for each group. * indicates p < 0.05 vs control, # indicates p < 0.05 vs ISO.

Loss of pro- and anti-oxidant balance in the cell leads to oxidative stress and cellular injury. ISO is widely known to induce oxidative stress by enhancing ROS and RNS production while reducing endogenous antioxidant activity. Therefore, we examined if canagliflozin treatment is effective against ISO-induced oxidative and nitrative stress. Agreeing with previous reports, we found that ISO treatment significantly elevated levels of oxidative and nitrative stress markers such as MDA, NO, MPO and APOP in kidney tissue homogenates (Fig. 1A–D). In addition, plasma levels of MDA, NO and APOP were also increased in ISO treated animals, reflecting tissue damage and subsequent leakage of these markers into the plasma (Fig. 1A,B,D). Due to confounding factors such as hemolysis, heme-associated proteins and peroxidases that modulate MPO in the plasma, plasma MPO was not assessed. Importantly, canagliflozin treatment in ISO rats produced a sharp decrease in those oxidative/nitrative markers in kidney tissue as well as in the plasma (Fig. 1A–D). These data indicate that canagliflozin has significant in vivo antioxidant action.

### Canagliflozin treatment boosts endogenous antioxidant defense

**Figure 2:**
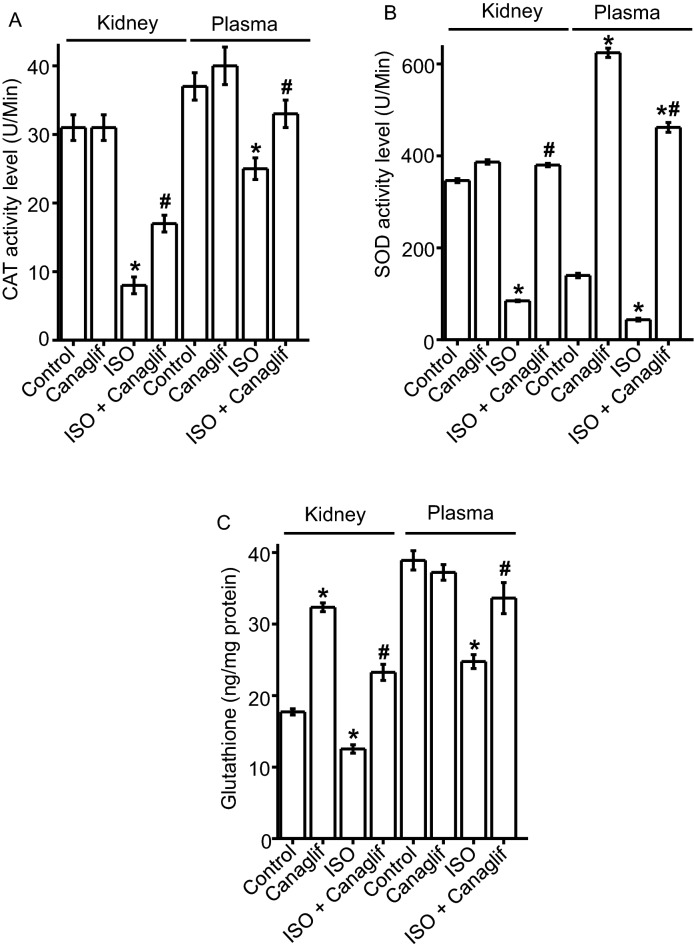
Canagliflozin (Canaglif) treatment rescues ISO-induced reduction of endogenous antioxidants. Statistical significance was determined using one-way ANOVA with Newman–Keuls post hoc test. Data are presented as mean ± SEM. n = 5 for each group. * indicates p < 0.05 vs control, # indicates p < 0.05 vs ISO.

ISO-treatment produces oxidative stress by enhancing ROS and RNS levels while simultaneously reducing naturally occurring endogenous antioxidants. We therefore examined the effects of canagliflozin on the activity of endogenous antioxidants such as SOD, CAT and glutathione. We found a significant reduction in SOD and CAT enzyme activity and glutathione levels in the plasma and kidney tissue homogenate in ISO-treated rats compared to their corresponding controls (Fig. 2A–C). Importantly, canagliflozin treatment restored SOD and CAT activities, and prevented glutathione reduction in plasma and kidney tissue homogenate from ISO-treated rats (Fig. 2A–C). These data demonstrate that canagliflozin enhances endogenous antioxidant defenses in kidney and in plasma.

### Canagliflozin stimulates phosphorylation of AMPK, Akt and eNOS

**Figure 3:**
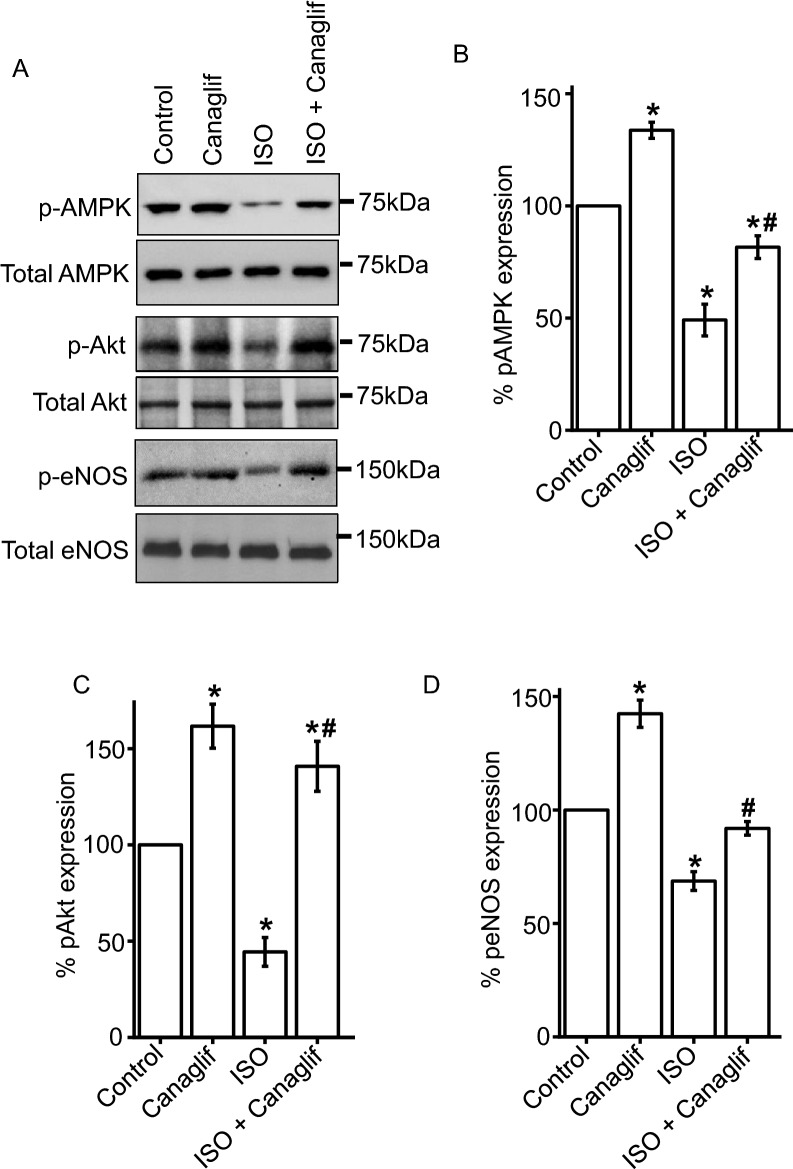
Canagliflozin (Canaglif) stimulates AMPK, Akt and eNOS phosphorylation. (**A**) Representative Western blot images of phosphorylated (p) proteins normalized to their total protein expression for AMPK, Akt and eNOS. Full-length blot images are presented in supplementary figure 1. (**B**–**D**) Mean data comparing relative changes of phosphorylation normalized to total protein expression. One-way ANOVA with Newman–Keuls post hoc test was used for multiple group comparisons. Data are presented as mean ± SEM. n = 4–5 for each group. * indicates p < 0.05 vs control, # indicates p < 0.05 vs ISO.

Our biochemical data demonstrate that canagliflozin possesses strong antioxidant and anti-inflammatory properties. We therefore attempted to identify the underlying antioxidant and anti-inflammatory signaling mechanisms promoted by canagliflozin. Given that the AMPK–Akt–eNOS signaling axis has been shown to have protective anti-inflammatory actions in various cardiovascular diseases^14,15^, we hypothesized that canagliflozin may activate this signaling cascade. Our Western blotting data demonstrate that ISO treatment attenuates phosphorylation, and hence activation, of AMPK at Thr172, Akt at Ser473 and eNOS at Ser1177, respectively. This effect is significantly reversed by canagliflozin treatment (Fig. 3A–D). Importantly, treatment with canagliflozin alone also significantly increases AMPK, Akt and eNOS phosphorylation (Fig. 3A–D). In summary, our data suggest that canagliflozin stimulates the AMPK–Akt–eNOS signaling axis, which may reduce oxidative stress and inflammation, and provide renoprotection.

**Figure 4:**
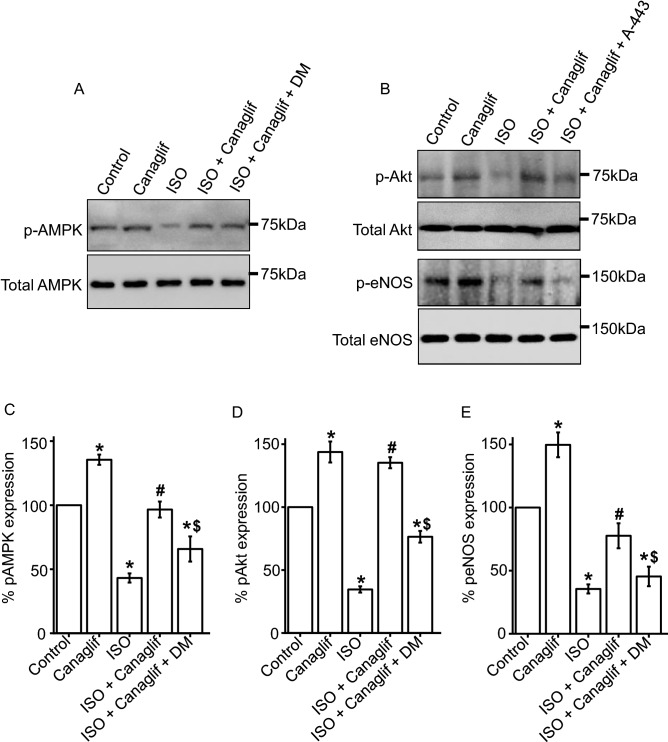
Canagliflozin stimulates AMPK, Akt and eNOS phosphorylation in human renal proximal tubule epithelial cells. (**A**,**B**) Representative Western blot images of phosphorylated (p) proteins normalized to their total protein expression for AMPK, Akt and eNOS. Full-length images are presented in supplementary figure 2. (**C**–**E**) Mean data comparing relative changes of AMPK, Akt and eNOS phosphorylation normalized to total protein expression. DM and A-443 indicate dorsomorphin and A-443654, respectively. One-way ANOVA with Newman–Keuls post hoc test was used for multiple group comparisons. Data are presented as mean ± SEM. n = 4 for each group. * indicates p < 0.05 vs control, # indicates p < 0.05 vs ISO, $ indicates p < 0.05 vs ISO + Canaglif.

To further validate the involvement of the AMPK–Akt–eNOS axis, we used pharmacological inhibitors of this signaling axis in human primary renal proximal tubule epithelial cells. Consistent with our previous results above, ISO treatment reduces AMPK phosphorylation, which is reversed by canagliflozin treatment. Inhibition of AMPK with dorsomorphin reverses canagliflozin-mediated rescue of AMPK phosphorylation, further supporting the role of canagliflozin in influencing this signaling cascade (Fig. 4A,C). Similarly, A-443654, an inhibitor of Akt, significantly reduces the canagliflozin-mediated increase in Akt phosphorylation (Fig. 4B,D). Since Akt is an upstream regulator of eNOS, we next examined the hypothesis that Akt inhibition by A-443654 may reduce eNOS phosphorylation. Our data shows that A-443654 blocks canagliflozin-mediated reversal of eNOS phosphorylation (Fig. 4B,E). Taken together, our data demonstrate that canagliflozin enhances phosphorylation and activation of AMPK, Akt and eNOS, which may underlie the previously shown renoprotective actions against ISO-induced oxidative stress.

### Canagliflozin suppresses ISO-induced NOX4 and iNOS upregulation

**Figure 5:**
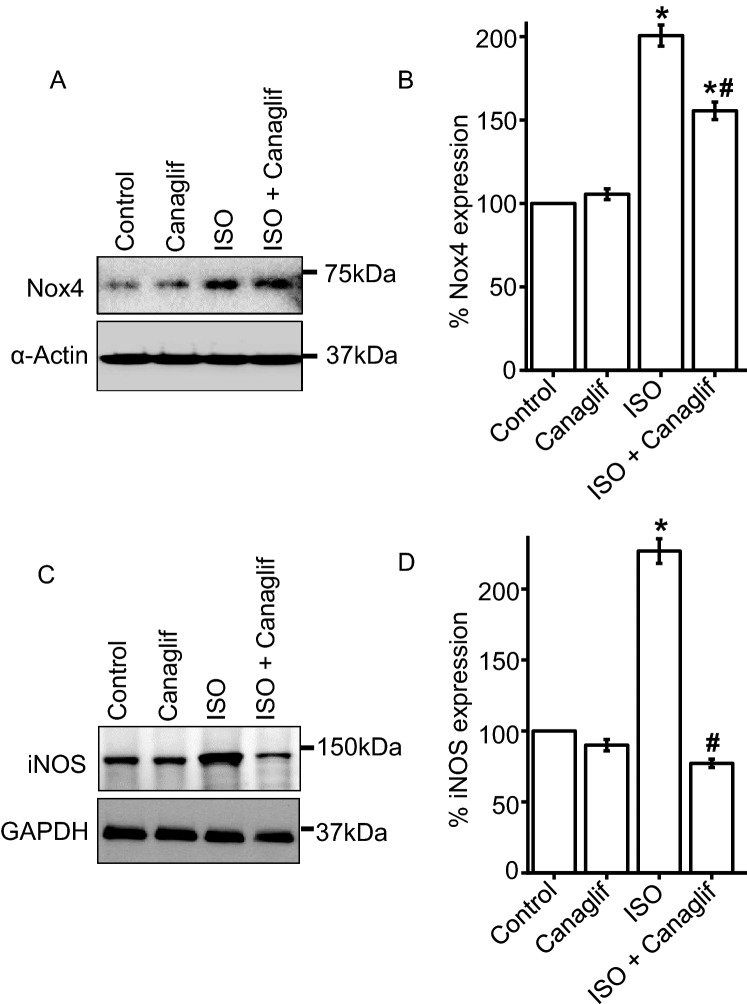
Canagliflozin (Canaglif) reduces ISO-mediated upregulation of NOX4 and iNOS. (**A**) Representative Western blot image showing a reduction of Nox4 protein expression by canagliflozin. Full-length Western blot images are presented in supplementary figure 3. (**B**) Mean data for changes of Nox4 expression normalized to α-actin. n = 4 for each group. (**C**) Representative Western blot image showing a reduction of ISO-mediated iNOS upregulation by canagliflozin treatment. (**D**) Mean data for changes of iNOS expression normalized to α-actin or GAPDH expression. One-way ANOVA with Newman–Keuls post hoc test was used for multiple group comparisons. Data are presented as mean ± SEM. n = 4 for each group. * indicates p < 0.05 vs control, # indicates p < 0.05 vs ISO.

ISO-treatment has previously been shown to increase the expression of NOX4 (NADPH oxidase isoform 4) and stimulate superoxide radical production, resulting in oxidative stress. Therefore, we examined the potential involvement of Nox4 upregulation and resultant oxidative stress in our model. ISO treatment caused a ~ 2-fold increase in NOX4 protein expression (Fig. 5A,B) compared to controls. In contrast, canagliflozin treatment reduced ISO-associated Nox4 overexpression by ~ 23% (Fig. 5A,B).

Regulated NO production by eNOS is essential for cardiovascular homeostasis, whereas inflammation promotes upregulation of inducible nitric oxide synthase (iNOS), which facilitates nitrative stress by producing up to 1,000-fold more NO than eNOS. Studies have reported that Nox4 signaling is an upstream inducer of iNOS^16^, and given that ISO treatment elevates NO levels (Fig. 1B), we investigated the effects on iNOS expression. Indeed, our results demonstrate that iNOS expression sharply increases (~ 2.3-fold) in kidney of ISO-treated rats (Fig. 5C,D), an effect that was significantly reduced (~ 66%) by canagliflozin (Fig. 5C,D). Taken together, our data suggest that canagliflozin suppresses ISO-associated iNOS upregulation, perhaps by reducing NOX4 expression and consequent inhibition of the redox signaling required for iNOS induction.

### Canagliflozin attenuates ISO-induced kidney cell apoptosis

**Figure 6:**
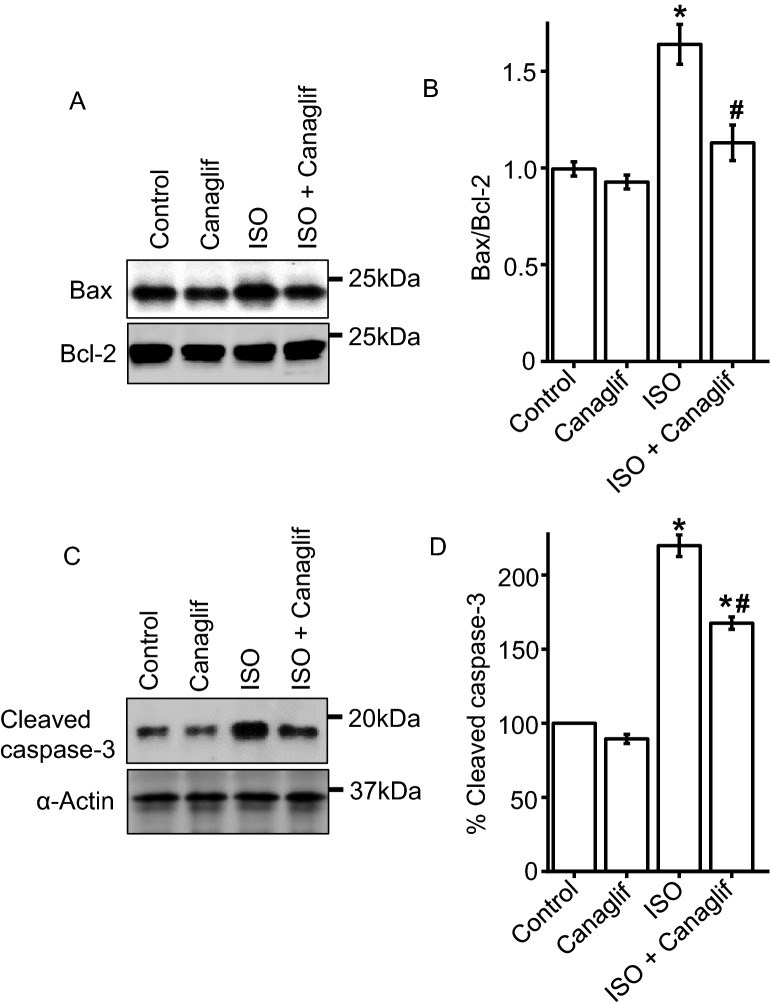
Canagliflozin (Canaglif) treatment attenuates apoptosis of kidney cells by ISO. (**A**) Representative Western blot image illustrating a reduction of Bax protein expression by canagliflozin. Full-length blot images are presented in supplementary figure 4. (**B**) Mean data for changes of Bax protein levels relative to anti-apoptotic protein Bcl-2. (**C**) Example Western blot image showing that canagliflozin treatment lowered cleaved caspase-3 expression. (**D**) Mean data comparing relative changes of cleaved caspase-3 protein expression. One-way ANOVA with Newman–Keuls post hoc test was used for multiple group comparisons. Data are presented as mean ± SEM. n = 4 for each group. * indicates p < 0.05 vs control, # indicates p < 0.05 vs ISO.

Previously, AMPK and Akt activation were reported to reduce stress-induced apoptosis of renal tubular cells in vitro and attenuation of cardiomyocyte apoptosis due to myocardial IRI in vivo^17,18^. In line with previous reports, our data demonstrate that canagliflozin stimulates phosphorylation and activation of both AMPK and Akt. Therefore, we asked if AMPK–Akt activation could attenuate oxidation-induced apoptosis of kidney cells. To do so, we analyzed expression levels of the pro-apoptotic protein Bax and anti-apoptotic protein Bcl-2, whereby a ratio of < 1.0 Bax to Bcl-2 (Bax/Bcl-2) protein indicates cell viability, and a ratio > 1.0 indicates apoptosis. Our data indicate that the ratio of Bax to Bcl-2 protein expression in control and canagliflozin treated animals were ~ 0.99 and ~ 0.92, respectively (Fig. 6A,B). However, in ISO-treated conditions, this increases to ~ 1.63. Treatment with canagliflozin markedly reduces the Bax/Bcl-2 ratio to ~ 1.13 (Fig. 6A,B), demonstrating renoprotection from ISO-induced apoptosis.

To further explore the anti-apoptotic actions of canagliflozin, we analyzed the expression of the apoptotic-marker cleaved caspase-3, which is elevated upon pro-apoptotic signaling and programmed cell death^19^. Our data demonstrate that control and canagliflozin-treated rat tissues had low cleaved caspase-3 expression, indicative of basal caspase-3 activation and apoptosis (Fig. 6C,D). However, ISO- treated rats exhibit significantly elevated cleaved caspase-3 levels (~ 220% of control). Canagliflozin treatment partially, but significantly, reversed the ISO-induced effect to ~ 167%. In summary, our data indicate that canagliflozin attenuates oxidative stress-induced apoptosis of kidney cells by regulating levels of pro-apoptotic Bax and anti-apoptotic Bcl-2 expression, and inhibiting caspase-3 activation, likely via modulation of AMPK and Akt signaling.

### Canagliflozin treatment reduces inflammation and fibrosis and improves renal function

**Figure 7:**
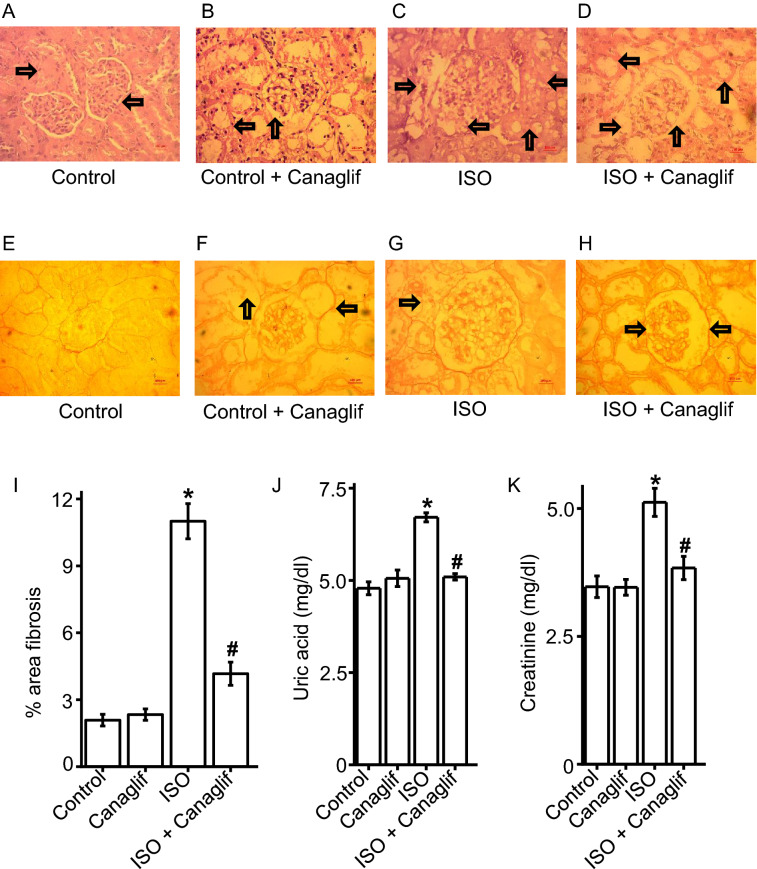
Canagliflozin (Canaglif) treatment reduces inflammation and fibrosis and improves kidney function. (**A**–**D**) Representative images of H&E staining showing presence or absence of inflammatory cell infiltration in kidney tissue sections under different conditions. (**E**–**H**) Representative images of picrosirius red staining showing presence and/or extent of fibrosis in kidney tissue sections. (**I**) Mean data illustrating % of area fibrosis under different conditions, n = 12 for each group. (**J**,**K**) Mean data showing that canagliflozin abolished elevation of renal function markers uric acid and creatinine in the plasma. One-way ANOVA with Newman–Keuls post hoc test was used for multiple group comparisons. Data are presented as mean ± SEM. n = 5 for each group. * indicates p < 0.05 vs control, # indicates p < 0.05 vs ISO.

While oxidative stress and downstream inflammation cause renal injury, the abundance of polyunsaturated fatty acids in kidneys makes them particularly susceptible to ROS and RNS damage and inflammation^18^. Our results demonstrate that canagliflozin possesses in vivo antioxidant properties. We therefore investigated if canagliflozin can protect against kidney damage by reducing inflammation secondary to ISO-mediated ROS/RNS generation. We examined the effects of canagliflozin on inflammatory cell infiltration and fibrosis by performing H&E staining and picrosirius red staining on kidney tissue sections. H&E staining images show that kidney sections from control and canagliflozin-treated animals maintain normal histoarchitecture (Fig. 7A,B), whereas sections from ISO-treated animals exhibit a significant increase in inflammatory cell infiltration and necrotic changes. Importantly, these effects are strongly suppressed upon canagliflozin treatment (Fig. 7C,D). Similarly, picrosirius red staining of control and canagliflozin-treated rat kidney tissue sections shows normal collagen distribution and alignments, indicating an absence of fibrosis (Fig. 7E,F,I). Tissue from ISO-treated rats, exhibited excessive collagen deposition and fibrosis, which was significantly reduced upon canagliflozin treatment (Fig. 7G–I). Collectively, our data indicate that canagliflozin treatment attenuates ISO-induced inflammation and fibrosis of kidney tissues.

Oxidative stress leads to kidney injury that is commonly accompanied by elevation of plasma uric acid and creatinine; two widely used clinical kidney function markers. As our results demonstrate that canagliflozin possesses potent antioxidant, anti-inflammatory and anti-apoptotic effects, we investigated whether canagliflozin influences these markers of renal function. Our data show that ISO-treated rats have significant increases in plasma uric acid and creatinine levels when compared to controls (Fig. 7J,K). Importantly, canagliflozin treatment prevented the elevation of both uric acid and creatinine levels, indicating an improvement of kidney function. In summary, our data demonstrate that canagliflozin augments kidney function and provides renoprotection in ISO-treated rats by stimulating antioxidant, anti-inflammatory and anti-apoptotic signaling, to counter the deleterious effects of ROS/RNS during ISO-induced oxidative stress.

## Discussion

Our study demonstrates, for the first time, that canagliflozin treatment protects against kidney damage in an ISO-induced oxidative stress rat model that mimics the pathological features of oxidative kidney injury in humans, which arise due to excessive SNS activity often seen in diabetes^18,20^. We found that canagliflozin treatment augmented endogenous antioxidant defense and reduced oxidative stress markers, inflammation, fibrosis and apoptosis. Furthermore, canagliflozin treatment stimulated antioxidant/anti-inflammatory signaling pathways involving AMPK, Akt and eNOS, while inhibiting pro-oxidative/pro-inflammatory and pro-apoptotic signaling involving iNOS, Nox4, Bax, and caspase-3 activation. Consistent with improved biochemical and molecular markers, canagliflozin treatment produced significant improvement of kidney function in ISO-treated rats.

In diabetes, autonomic neuropathy and vagal impairment lead to SNS overstimulation that contributes to chronic kidney disease^2^. It is well recognized that due to its non-specific action on β1- and β2-AR, both of which are present in kidney tissue, ISO treatment mimics SNS overstimulation, resulting in oxidative stress, inflammation and consequent tissue damage^5,11,21,22^. Previous studies attributed ISO-induced oxidative stress to increased production of oxidative and nitrative stress markers such as MDA, NO, MPO and APOP, as well as concurrent reduction of endogenous scavenger antioxidants such as SOD, CAT and glutathione^23,24^, corroborating findings in our current study. Importantly, canagliflozin treatment strongly suppressed oxidative stress in ISO rats by reducing oxidative stress markers such as MDA, NO, MPO and APOP; and by elevating antioxidant contributors such as SOD, CAT and glutathione. The observed antioxidant action of canagliflozin may extend beyond kidneys to a systemic level, as evidenced by the upregulation of antioxidants and simultaneous reduction of stress markers in the plasma. Our Western blotting data demonstrated that canagliflozin strongly suppresses ISO-induced overexpression of Nox4, an important trigger for superoxide radical production, which leads to oxidative stress and inflammation. Apart from its direct contribution to oxidative stress, Nox4 is also known to induce iNOS that stimulates nitrative stress via excessive NO production as seen in ISO-treated rat tissue and plasma samples in our study. Oxidative and nitrative stress facilitate tissue inflammation and fibrosis, and consistent with this, our H&E and picrosirius red staining confirmed that ISO-treated rat kidney tissues had increased oxidative/nitrative stress markers, inflammatory cell infiltration, collagen deposition and fibrosis (Fig. 7). Meanwhile, our histochemical results revealed that ISO-treated rat tissues had significantly high inflammatory cell infiltration and increased collagen deposition, which correlates with observed iNOS upregulation and nitrative stress in the ISO animal kidney tissues. Canagliflozin treatment reversed ISO-induced inflammatory and fibrotic changes in kidney tissues, which is indicative of its protective role against oxidative stress, inflammation and fibrosis caused by ISO-induced oxidation. Importantly, canagliflozin reduced both ISO-induced Nox4 and iNOS upregulation, leading us to propose that the antioxidant/anti-inflammatory action of canagliflozin partially lies in its ability to suppress Nox4 and downstream iNOS induction. However, whether canagliflozin-mediated reduction of Nox4 and iNOS takes place in series or in parallel remains to be determined. Although iNOS-stimulated excessive NO production results in nitrative stress and cellular injury, eNOS-mediated NO production is essential for physiological function and is protective against oxidative damage.

We found that canagliflozin not only inhibited pro-oxidative and inflammatory signaling mediated by Nox4 and iNOS but also stimulated a critical protective signaling axis involving AMPK, Akt and eNOS in the kidney tissues. This is in agreement with a previous report by Lieberthal et al. demonstrating that AMPK activation and downstream PI3K and Akt signaling in mice renal tubular cells attenuated oxidative stress and tubular cell death in ischemic kidney injury^14,15^. Previous studies report that canagliflozin stimulates AMPK signaling in cardiomyocytes^17^ and endothelial cells^25^, producing a range of protective effects in the cardiovascular system^26^, such as anti-inflammatory actions and protection against myocardial ischemia^17^. Canagliflozin-induced activation of Akt was shown to protect against myocardial IRI^17^. Importantly, activated AMPK and Akt phosphorylate the eNOS-Ser1177 activation site, which in turn stimulates NO production. Several studies reported the role of eNOS-mediated NO signaling against oxidative stress, apoptosis, and platelet aggregation following canagliflozin administration^17,19^. AMPK–Akt signaling is believed to prevent apoptosis following myocardial IRI^17,27,28^. Moreover, this study demonstrated that canagliflozin treatment reversed apoptosis of kidney cells by inhibiting Bax and caspase-3 activation. Such anti-apoptotic action of canagliflozin may be mediated through the activation of AMPK–Akt signaling axis^15^. The overall reduction of ISO-mediated oxidative stress, inflammation and apoptosis of renal tubular cells by canagliflozin is likely due to combined effects of preserved NO signaling as well as reduction of Nox4, iNOS, Bax and caspase-3 activation. Since SNS overstimulation and resultant diabetic nephropathy is prevalent in diabetic patients, who are often left with limited options for treating this condition, canagliflozin pharmacotherapy may provide an alternative option for the treatment of SNS hyperactivity-related kidney injury in diabetic patients.

Our data showed that canagliflozin treatment was effective in reducing both tissue and plasma oxidative stress markers and preserving the activity of endogenous antioxidants. Such findings further support the concept that SGLT2 inhibitors may have systemic antioxidant and anti-inflammatory actions beyond their localized effects on kidney transporters. Although the primary antidiabetic action of canagliflozin is due to a reduction of glucose reabsorption via blockade of SGLT2, this study also demonstrated that canagliflozin acts on multiple signaling pathways that are likely independent of its action on SGLT2. Therefore, the observed antioxidant, anti-inflammatory and anti-apoptotic actions of canagliflozin appear to be pleiotropic that may translate even beyond the kidneys^17,29^. In general, our data suggests that canagliflozin promotes multiple antioxidant/anti-inflammatory signaling pathways. However, other mechanisms not described here may also be responsible for the protective effects of canagliflozin against oxidative damage to the kidneys. Since SNS overstimulation and diabetic nephropathy is a common complication in diabetes, patients and diabetic nephropathy is associated with oxidative stress and inflammation of the renal system. Our study used ISO-induced oxidative stress rat model in non-diabetic rats, which is independent of diabetic specific pathological processes. Therefore, future studies on diabetic animal models will be required to understand full therapeutic potential of canagliflozin in SNS overstimulation-induced oxidative stress and organ injuries.

In summary, our study demonstrated that canagliflozin treatment has in vivo antioxidant, anti-inflammatory and anti-apoptotic actions that prevent kidney injury caused by ISO-induced oxidative stress. These actions may involve multiple mechanisms that may extend beyond localized actions in kidney tissues to broad systemic effects, and likely involve other organs that are affected by SNS hyperactivity such as blood vessels, heart and liver. Although, SNS hyperactivity and diabetic nephropathy are common in diabetic patients, conventional beta-blocker treatment is contraindicated due to a range of adverse effects. In this scenario, canagliflozin monotherapy may offer an attractive substitute.

## Materials and methods

### Chemicals and antibodies

Reduced glutathione, ethanol, dimethyl sulfoxide (DMSO), xylene, thiobarbituric acid, 10% neutral buffered formalin (NBF) solution, protease and phosphatase inhibitor cocktails were purchased from Sigma Aldrich (St. Louis, MO, USA). Canagliflozin and isoprenaline were from Square Pharmaceutical Ltd. (Dhaka, Bangladesh) and Samarth Life Sciences Pvt. Ltd. (Mumbai, India), respectively. All standards and assay components for MDA, NO, APOP assays, H&E (Hematoxylin and Eosin) and picrosirius red staining were from Merck (Darmstadt, Germany). Standard and SOD assay components were bought from SR Group (Delhi, India). CK-MB assay kit was purchased from DCI Diagnostics (Budapest, Hungary). Antibodies raised to AMPK, p-AMPK (Thr172), Akt, p-Akt (Ser473), p-eNOS (Ser1177) and cleaved caspase-3 were purchased from Cell Signaling Technology (Danvers, MA, USA), and those for eNOS were from Abcam (Cambridge, UK) and BD Biosciences (San Jose, CA, USA). Antibodies for α-actin, iNOS, Bax and Bcl-2, HRP-conjugated anti-mouse and anti-rabbit secondary antibodies were from Santa Cruz Biotechnology (Santa Cruz, CA, USA). Phosphate-buffered saline (PBS, pH 7.4), radioimmunoprecipitation (RIPA) buffer, blot stripping buffer (Restore) were bought from Thermo Fisher Scientific (Waltham, MA, USA). DC Protein Assay Kit, tween 20, SDS-PAGE gel components, protein molecular weight marker, 10 × tris-buffered saline (TBS) and PVDF (polyvinylidene fluoride) membrane were bought from Bio-Rad (Hercules, CA, USA).

### Animals and experimental design

The Ethics Committee of North South University approved all experimental protocols for animal care, handling and experimentation (AEC 005-2018). All experiments were performed in accordance with relevant guidelines and regulations. Twenty male Long Evans rats between 10 and 12 weeks of age were obtained from the Reproduction unit of the Animal House at North South University, Dhaka. All animals housed in individual cages in a temperature-controlled room (temperature 22 ± 2 °C; 55% humidity; 12-h light/dark cycles) had free access to standard chow diet and drinking water. The length of the experimental protocol was 2 weeks, where animals were randomly divided into four groups of five rats in each and treated as follows:

- Group I: Control—only received standard chow diet for the entire period of 2 weeks.
- Group II: Canagliflozin—given standard chow diet for the first week and then treated with canagliflozin at 5 mg/kg daily with chow diet for the second week^30^.
- Group III: ISO—given subcutaneous injections of isoprenaline at 50 mg/kg twice a week for the first week to induce oxidative stress^31^. Animals in this group received standard chow diet throughout the duration of the experiment.
- Group IV: ISO + canagliflozin—oxidative stress was induced first by subcutaneous injections of isoprenaline at 50 mg/kg twice a week for the first week and treated with canagliflozin at 5 mg/kg daily for the second week to study recovery.

### Euthanasia and tissue harvesting

Animals were euthanized by intraperitoneal injection of ketamine/xylazine (500/50 mg/kg) followed by decapitation. For plasma biochemistry, blood was drawn from the hepatic portal vein and plasma separated by spinning samples at 8,000 rpm for 15 min at 4 °C. Biochemical analyses were conducted on fresh plasma or stored at − 80 °C for future experiments. Kidneys were collected, weighed and processed for biochemical, Western blotting and histological examination and the remaining samples stored at − 80 °C.

### Plasma biochemistry

Uric acid and creatinine concentrations were determined using assay kits according to the manufacturer’s instructions (DCI Diagnostics, Budapest, Hungary).

### Quantification of oxidative and nitrative stress markers: assays for malondialdehyde (MDA), nitric oxide (NO) and advanced protein oxidation products (APOP)

Roughly, 0.1 g of kidney tissue from each group was placed in 1 ml of phosphate buffer (pH 7.4) in an Eppendorf tube, homogenized and centrifuged at 10,000 rpm for 15 min at 4 °C. Supernatants were transferred into fresh tubes and used for the determination of MDA, NO and APOP. Lipid peroxidation was expressed in terms of MDA levels in kidney tissue homogenates using a colorimetric assay^32^. NO concentration was measured following the method of Tracey et al.^33^, and was calculated by using a standard curve and expressed as nmol/g of tissue. APOP concentration was measured according to a modified protocol by Witko-Sarsat et al.^34^ and Tiwari et al.^35^ and concentration expressed as nmol·ml^−1^ chloramine-T equivalents.

### Determination of myeloperoxidase (MPO) activity

MPO activity was determined by an *o*-dianisidine-H_2_O_2_ method following protocols described previously by Rahman et al.^21^.

### Estimation of endogenous antioxidant levels: measurement of catalase (CAT) and super oxide dismutase (SOD) activity and glutathione (GSH) concentration

CAT activities in plasma and kidney tissue homogenate were determined as described elsewhere^36,37^. Absorbance was read at 240 nm, and an absorbance change of 0.01 units/min change was counted as one unit of CAT activity. SOD activity was measured according to protocols described previously^36,37^. Absorbance of the reaction mixtures containing enzymes was read at 480 nm for one minute at 15-s intervals. A blank without samples was run in parallel. Epinephrine auto-oxidation present in the assay system was calculated and 50% inhibition of epinephrine auto-oxidation is taken as one unit of SOD activity. Reduced glutathione level was determined following protocols described previously^38^. Absorbance of the reaction mixture was promptly read at 405 nm with the development of the yellow chromophore and glutathione level expressed as ng/mg protein.

### Histopathological examination of rat kidney sections

Kidney sections were fixed in 10% Neutral Buffered Formalin (NBF) followed by their treatment with graded ethanol and xylene. Sections were subsequently embedded into paraffin blocks and cut with a rotary microtome into 5 µm thin slices that were collected on fresh slides and stained with hematoxylin/eosin (H&E) to visualize immune cell infiltration in the tissue sections. Picrosirius red staining was also performed on the kidney sections in parallel to analyze the presence and extent of fibrosis. At the end of the staining procedure, slides containing stained sections were photographed and analyzed under a light microscope at 40 × magnification (Zeiss Axioscope)^21^. To quantify % of area fibrosis, ImageJ software (National Institutes of Health, Bethesda, MD) was used.

### Cell culture and treatment

Human primary renal proximal tubule epithelial cells were purchased from ATCC (PCS-400-010) and cultured according to the manufacturer’s instructions. Briefly, cells were maintained in epithelial cell culture medium (PCS-400-030, ATCC) supplemented with epithelial cell growth kit (PCS-400-040, ATCC) containing fetal bovine serum (0.5%), triiodothyronine (10 nM), rhEGF (10 ng/ml), hydrocortisone hemisuccinate (100 ng/ml), rh insulin (5 µg/ml), epinephrine (1 µM), transferrin (5 µg/ml) and l-Alanyl-l-Glutamine (2.4 mM). Cells were grown to approximately 80% confluency in the culture medium at 37 °C in a 95% air:5% CO_2_ humidified atmosphere. For our experiments, renal proximal tubule epithelial cells were treated as follows:

- Control: treated with vehicle (DMSO).
- Canaglif: treated with 10 µM canagliflozin for 12 h^25^.
- ISO: treated with 1 µM isoprenaline for 12 h^39^.
- ISO + Canaglif: co-treated with 1 µM isoprenaline and 10 µM canagliflozin for 12 h.
- ISO + Canaglif + DM: co-treated with 1 µM isoprenaline and 10 µM canagliflozin for 11 h, and then with 10 µM dorsomorphin^40^ for the last 1 h.
- ISO + Canaglif + A-443654: co-treated with 1 µM isoprenaline and 10 µM canagliflozin for 11.5 h, and then with 5 µM A-443654^41^ for the last 30 min.

At the end of the treatment protocol, adherent cells were briefly washed with ice-cold PBS. Cells were then solubilized in modified radioimmunoprecipitation (RIPA) buffer (50 mM Tris–HCl, 150 mM NaCl, 5 mM EDTA, 1% Nonidet P-40, 0.5% sodium deoxycholate, 0.1% SDS, 10 mM NaF, 10 mM Na_2_HPO_4_, pH 7.4) containing protease and phosphatase inhibitor cocktails. Cell lysates were processed for Western blotting as described below.

### Western blotting

To extract proteins from kidney tissues, approximately 0.1 g of kidney tissue was minced in an Eppendorf tube and homogenized in modified RIPA buffer. Tissue homogenates were then centrifuged at 12,000 rpm for 10 min at 4 °C and supernatants transferred into fresh tubes. Protein concentration was standardized using DC Protein Assay Kit, and about 50 µg protein for each sample was boiled with 2 × SDS-sample buffer (Bio-Rad), resolved by SDS-PAGE and blotted onto PVDF membrane. Membranes were blocked with 5% milk solution in TBST (tris-buffered saline with 0.1% Tween 20) for 1 h at room temperature and the incubated with the following primary antibodies overnight: total eNOS (1:500 dilution), p-eNOS (1:200 dilution), iNOS (1:500 dilution), α-actin (1:5,000 dilution), p-Akt (1:400 dilution), total Akt (1:500 dilution), p-AMPK-α (1:400 dilution), total AMPK-α (1:500 dilution), Bax (1:200), Bcl-2 (1:200) and cleaved caspase-3 (1:200 dilution). Membranes were washed and incubated with HRP–conjugated secondary antibodies (1:5,000 dilution) for 1 h at room temperature. Following secondary antibody incubation, blots were washed three times and developed, and protein bands imaged using Gel Doc XR + System (Bio-Rad). Densitometric analysis of the protein bands were performed using ImageJ software (64-bit Java 1.8.0_112, National Institutes of Health, Bethesda, MD; URL: https://imagej.nih.gov/ij/download.html). Where needed, blots were stripped of IgG using Restore stripping buffer and reprobed.

### Statistical analysis

Statistical analyses were carried out using OriginLab software v9.55 (2018b) (URL: https://www.originlab.com/index.aspx?go=SUPPORT&pid=3325). Values were expressed as mean ± standard error of mean (SEM). One-way analysis of variance (ANOVA) along with Newman–Keuls post-hoc test was used for multiple group comparisons. *p* < 0.05 (a priori) was considered statistically significant.

## Supplementary information

Supplementary Figures.
