# Supplementary material for: Canagliflozin ameliorates renal oxidative stress and inflammation by stimulating AMPK–Akt–eNOS pathway in the isoprenaline-induced oxidative stress model
Source: Sci Rep. 2020 Sep 4;10:14659. doi: 10.1038/s41598-020-71599-2 (PMC7474058; doi:10.1038/s41598-020-71599-2)
Supplement: Supplementary file 1 — Supplementary Figures. [file 41598_2020_71599_MOESM1_ESM.pdf]

Supplementary material

Canagliflozin ameliorates renal oxidative stress and inflammation by stimulating AMPK-Akt-eNOS pathway in the isoprenaline-induced oxidative stress model

Raquibul Hasan[a*], Shoumen Lasker[b], Ahasanul Hasan[a], Farzana Zerin[a], Mushfera Zamila[b], Faisal Parvez[b], Md. Mizanur Rahman[b], Ferdous Khan[b], Nusrat Subhan[b] and Md. Ashraful Alam[b*]

[a]Department of Pharmaceutical Sciences, College of Pharmacy, Mercer University, Atlanta, GA 30341, United States
[b]Department of Pharmaceutical Sciences, North South University, Dhaka 1229, Bangladesh

***Correspondence:**
Dr Md. Ashraful Alam, Associate Professor, Department of Pharmaceutical Sciences, North South University, Bashundhara, Dhaka, 1229, Bangladesh.
E-mail: ashraful.alam@northsouth.edu

And

Dr Raquibul Hasan, Assistant Professor, Department of Pharmaceutical Sciences, College of Pharmacy, Mercer University, 3001 Mercer University Drive, Atlanta, GA 30341, United States.
E-mail: hasan_r@mercer.edu

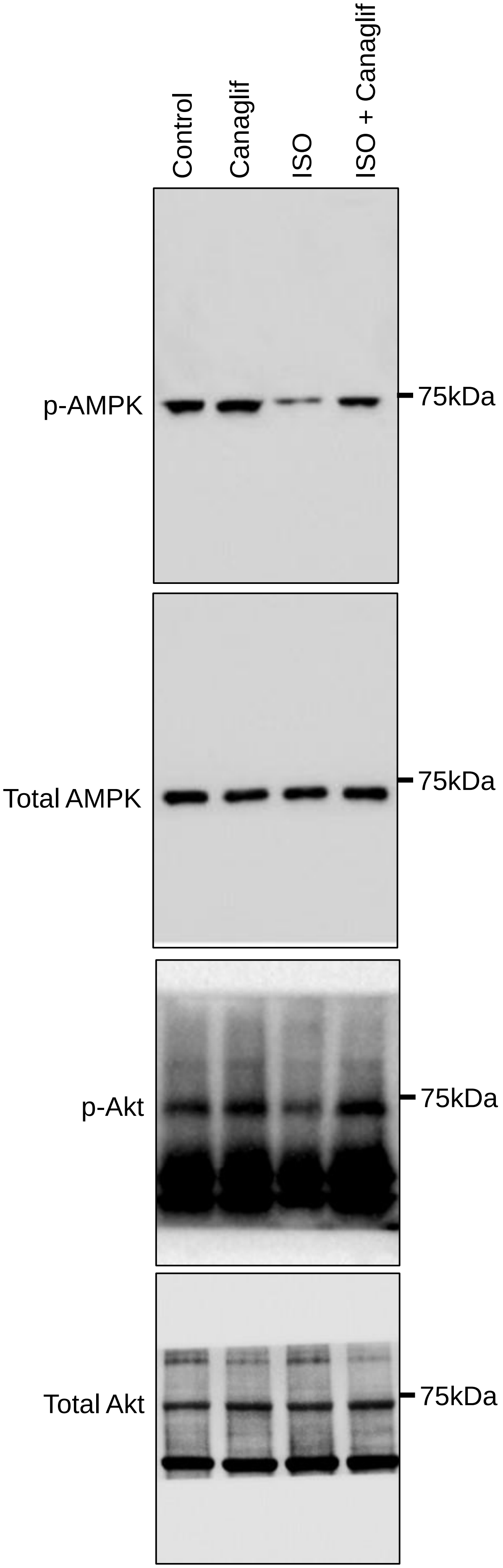

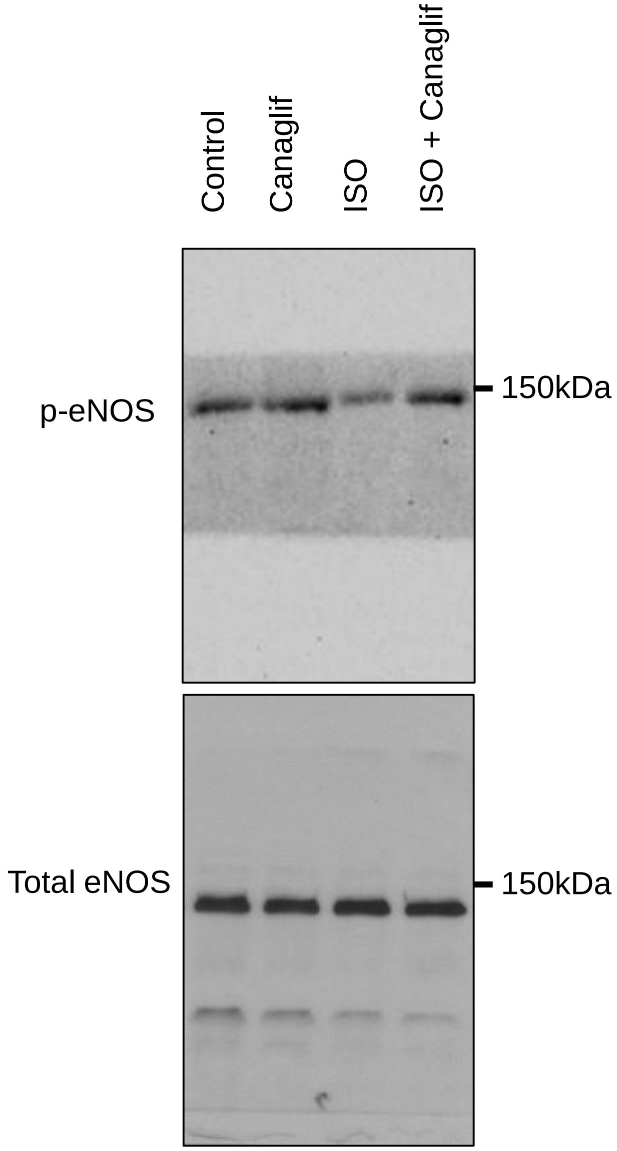

Supplementary Figure 1: Full-length Western blot images for p-AMPK, total AMPK, p-Akt, total Akt, p-eNOS and total eNOS representing Western blot images in Figure 3.

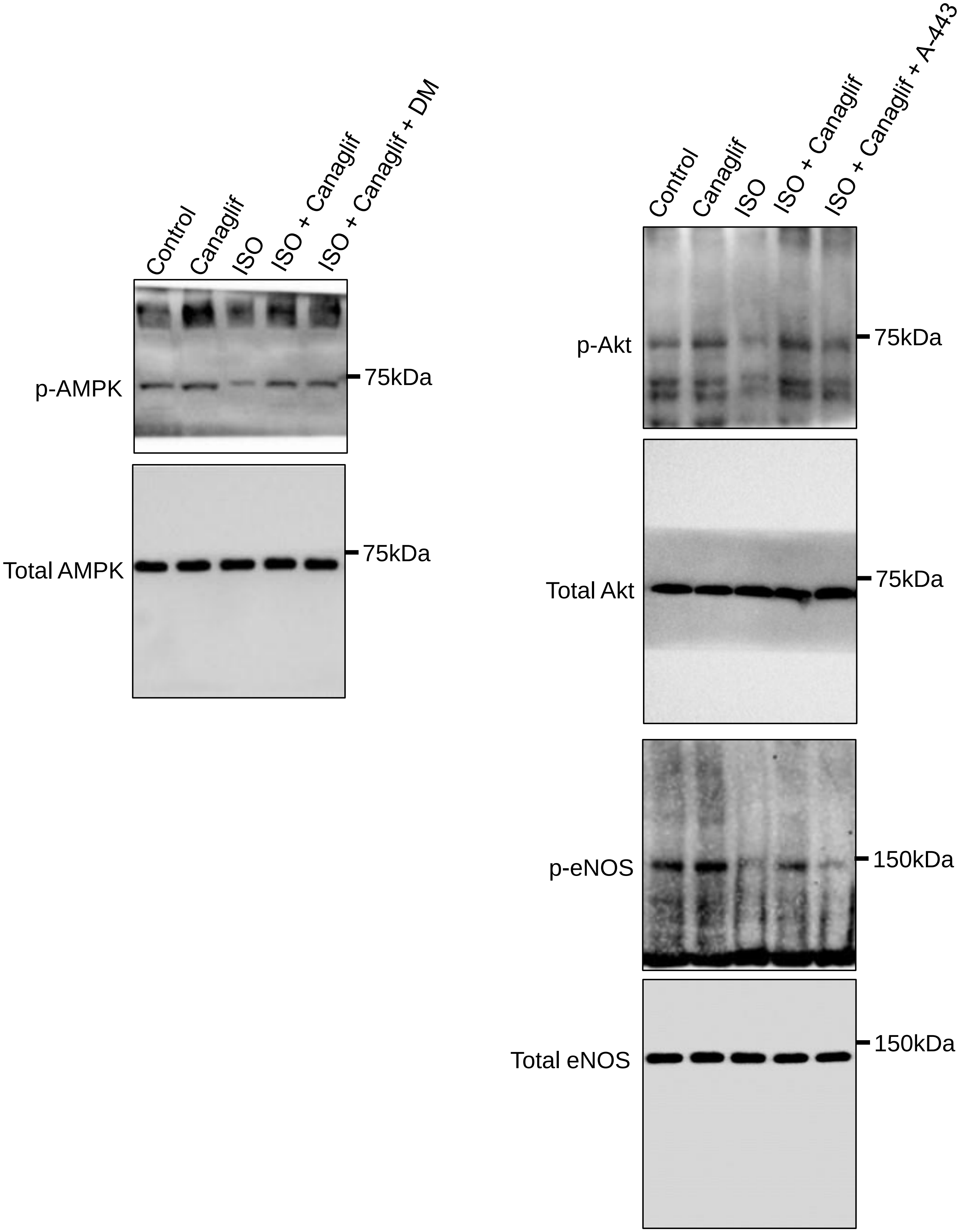

Supplementary Figure 2: Full-length Western blot images for p-AMPK, total AMPK, p-Akt, total Akt, p-eNOS and total eNOS representing Western blot images in Figure 4.

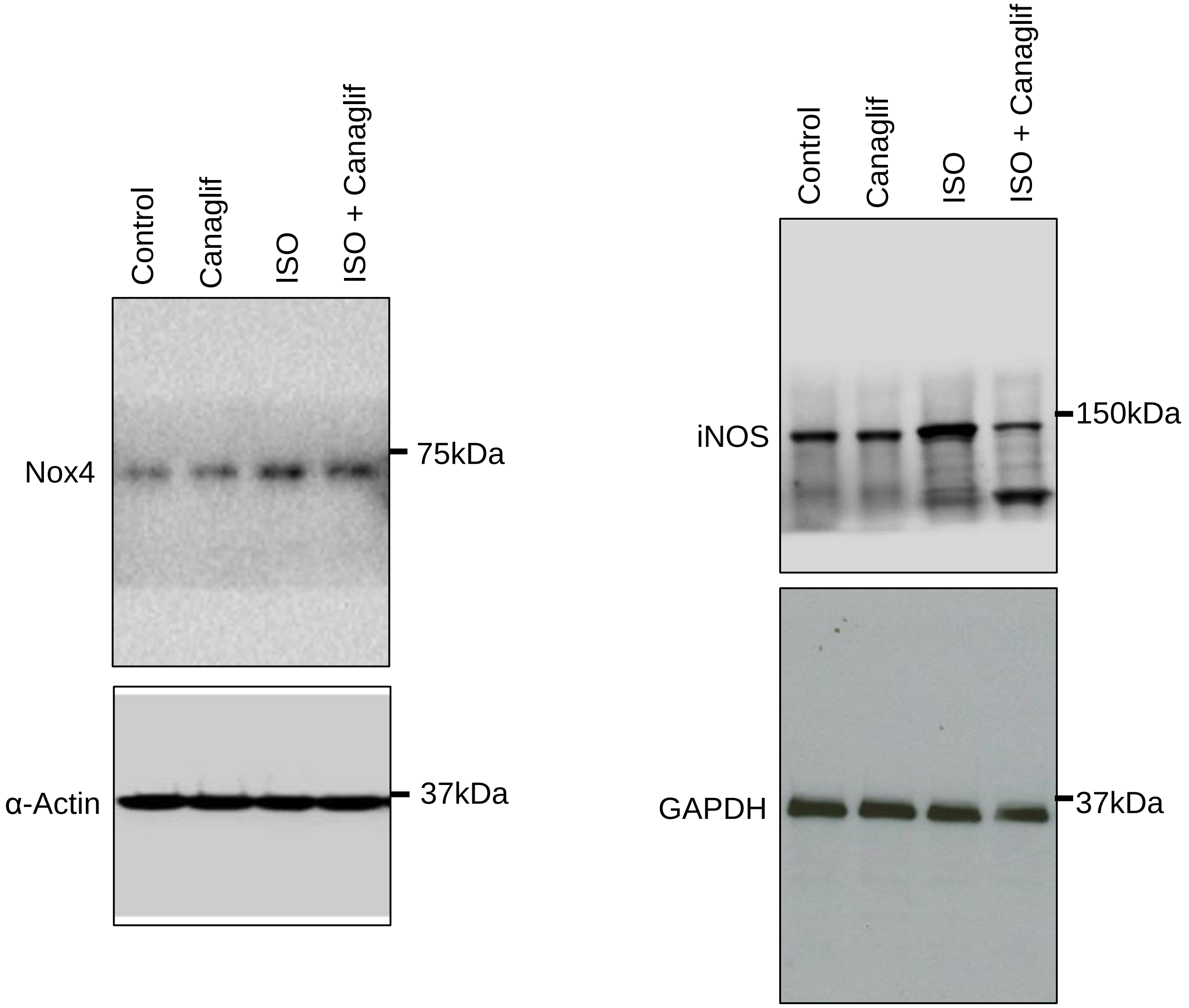

Supplementary Figure 3: Full-length Western blot images for Nox4, α-Actin, iNOS, GAPDH representing Western blot images in Figure 5.

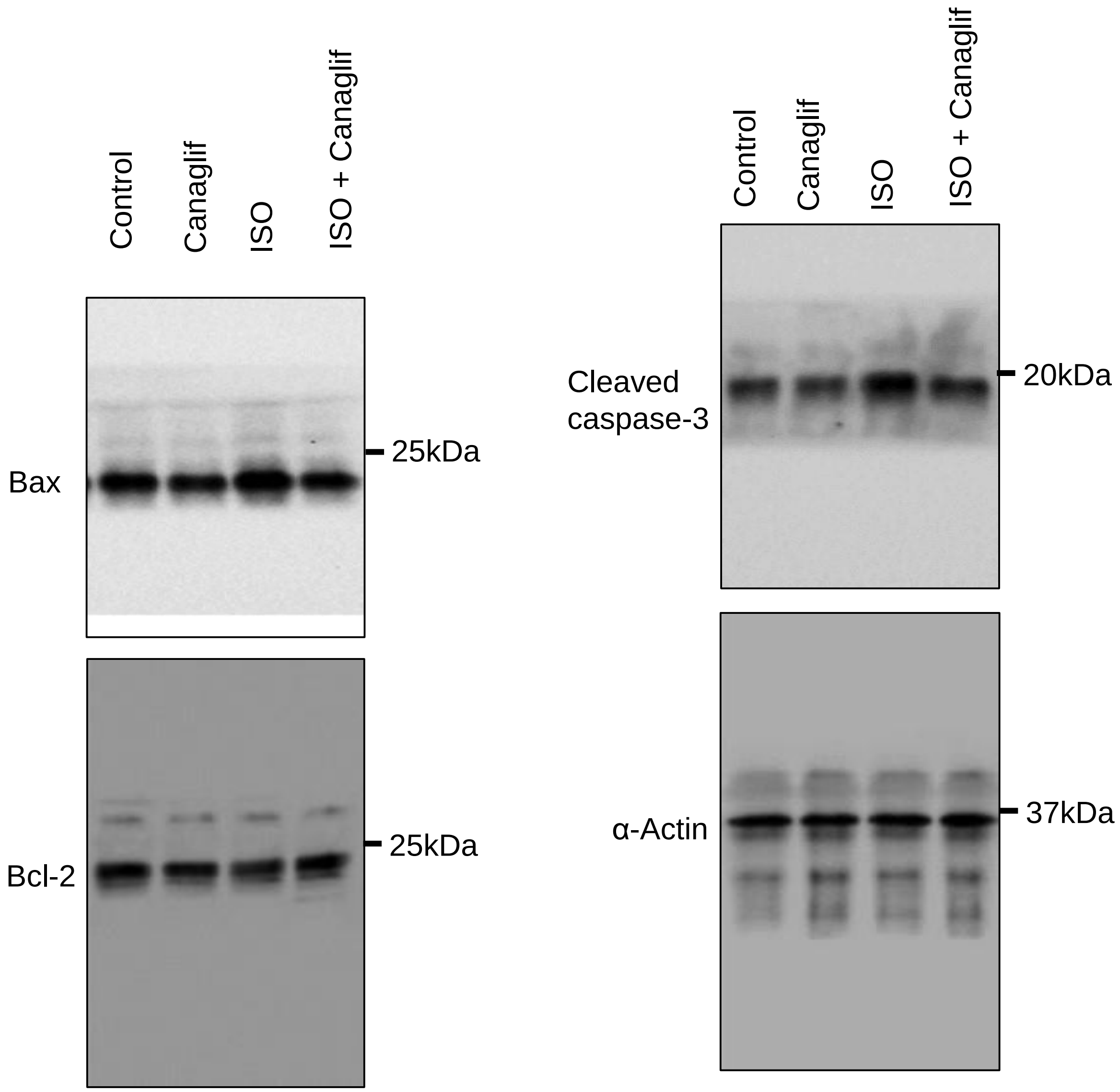

Supplementary Figure 4: Full-length Western blot images for Bax, Bcl-2, Cleaved caspase-3 and α-Actin representing Western blot images in Figure 6.
